# Model-Driven Deep Learning Enables Speckle-Free Holography for 3D Parallel Nanofabrication

**DOI:** 10.34133/research.1159

**Published:** 2026-04-14

**Authors:** Kexuan Liu, Wenqi Ouyang, Chuxian Chen, Jiachen Wu, Liangcai Cao, Shih-Chi Chen

**Affiliations:** ^1^ Department of Precision Instrument, Tsinghua University, Beijing, 100084, China.; ^2^ Department of Mechanical and Automation Engineering, The Chinese University of Hong Kong, Hong Kong, 999077, China.; ^3^ Centre for Perceptual and Interactive Intelligence, Hong Kong Science Park, Shatin, N.T., Hong Kong, 999077, China.

## Abstract

Holographic light fields offer a promising route toward high-throughput 3-dimensional (3D) nanofabrication. However, the fabrication uniformity remains limited by severe speckle noise present in many previously demonstrated hologram coding methods. Here, we address this challenge by developing a model-driven deep learning framework that enables speckle-free hologram generation with high uniformity. We demonstrate its practical feasibility for 3D nanofabrication using 2-photon lithography (TPL) and term this approach SMART HoloTPL. By establishing a polymerization model based on the broadband angular-spectrum method, self-supervised network training is guided to explore advanced hologram coding strategies without reliance on paired datasets. Tailored neural network architecture and loss functions are designed for high-uniformity hologram generation. A TPL fabrication platform powered by a femtosecond regenerative laser amplifier has been built. SMART HoloTPL achieves large-scale speckle-free 3D nanofabrication with a 120,000 voxels/s throughput and 120-nm resolution, addressing key challenges in fabrication rate and quality.

## Introduction

Compared with conventional laser sources, the femtosecond (fs) laser, featuring an ultra-short pulse width and extremely high peak power, results in minimal thermal effects and wide material applicability, thus emerging as a powerful tool for 3-dimensional (3D) micro/nanofabrication [1]. In particular, 2-photon lithography (TPL) relies on the simultaneous absorption of 2 near-infrared photons within a tightly focused fs-laser spot to trigger nonlinear polymerization, allowing in-volume true 3D micro/nanofabrication with subdiffraction-limit resolution [2,3]. TPL has been widely applied in micro-optics [4–6], micro-electro-mechanical system (MEMS) [7], microfluidics [8], microrobots [9–11], biomedicine [12], etc. However, state-of-the-art commercial TPL systems perform 3D printing in a voxel-by-voxel serial scanning approach, limiting the fabrication rate to ~0.1 mm^3^/h at ~150-nm resolution. This greatly reduces the manufacturing efficiency and compromises the yield of fabricated structures, preventing TPL from large-scale industrial applications.

With the advent of regenerative laser amplifiers, the peak pulse energy of fs lasers has increased by orders of magnitude. Consequently, parallelization in TPL has become feasible, yielding a substantial increase in fabrication throughput. Among various parallel TPL techniques, holography-based TPL, which integrates TPL with computer-generated holography (CGH), holds a promising route [13]. CGH enables 3D light field modulation and has a high degree of design freedom compared with structured light fields [14–16]. Specifically, CGH encodes target light fields into holograms by iterative algorithms such as the Gerchberg–Saxton (GS) algorithm [17]. The incident laser is modulated by a spatial light modulator (SLM) loaded with the hologram. The target light fields are then reconstructed by diffraction propagation or Fourier transform. Holography-based TPL utilizes demagnified light fields to realize multi-focus [18–23], layer-by-layer [24–26], and volumetric photopolymerization [27–29]. However, long-standing speckle noise compromises the quality and controllability of this approach, especially when the foci are closely spaced or form continuous patterned focal fields [24–27,30,31]. This limitation arises from existing hologram coding methods reliant on iterative algorithms.

Several strategies have been proposed to mitigate the nonuniformity caused by speckle noise. First, a multi-exposure approach [25] and a time-multiplexing approach [30,31] trade fabrication throughput for noise averaging. Meanwhile, modified GS algorithms—particularly those using a smoothly varying initial phase (e.g., a conical gradient)—achieve improved speckle suppression in 2D planar polymerization [26]. Achieving truly speckle-free volumetric fabrication requires sophisticated light field tailoring, such as focal field engineering (FFE) [27–29]. This is because the GS algorithm is an alternating projection method, which inherently tends to stagnate at a local optimum with limited reconstruction fidelity. More recently, deep learning technologies have revitalized various fields of optics, surpassing the convergence and quality limitations of classical approaches. Although some representative deep-learning-based CGH approaches have been reported [32–35], they mainly aim at enhancing holographic display quality and therefore are not suitable for holography-based TPL applications.

In this work, we present a speckle-free model-driven artificial intelligence holography-based TPL, dubbed SMART HoloTPL. We formulate the hologram generation process for SMART HoloTPL as an inverse problem governed by a known physical forward model and solve it using a model-driven deep learning framework. Specifically, a polymerization model based on the broadband angular-spectrum method (ASM) is established to guide a self-supervised neural network in optimizing hologram coding strategies. The network architecture is tailored to meet the demands of high-fidelity and large-scale hologram generation. The intensity and uniformity of the modulated light fields are constrained by a composite loss function. Powered by a fs regenerative laser amplifier with sufficient pulse energy, SMART HoloTPL achieves speckle-free parallel 3D fabrication with 120-nm resolution and 120,000 voxels/s.

## Results

### Origins of speckle noise in holography-based TPL

Hologram generation in holography-based TPL is an inverse problem with a known physical forward model. Both ill-posed target intensity distributions and practical hardware limitations constrain the calculation of holograms. Current SLMs impose constraints on holograms, including limitations to either amplitude or phase modulation, finite bandwidth, and finite pixel number. Therefore, hologram generation is a nonconvex inverse problem with non-unique, unstable, and potentially non-existent solutions [36]. Alternating projection algorithms, represented by the GS algorithm, are frequently applied to solve this problem [37,38]. The solution is gradually approximated through iterative projections between constraint sets, which is simple and effective. However, alternating projection algorithms tend to stagnate at a saddle point or local minima near the initial point and fail to achieve ideal convergence. To fully utilize the bandwidth of the SLM, a random phase is set as the initial point [39]. During the alternating projection process, positive and negative optical vortices are generated and annihilated in pairs until stabilized at a certain value, leading to convergence stagnation [39,40]. The initial random phase introduces mass optical vortices, exacerbating the impact of speckle noises in the modulated light fields.

In contrast, deep learning methods have recently shown robust and efficient capabilities in solving nonconvex optimization problems [32,41]. Firstly, benefiting from multilayer nonlinear structures and stochastic optimization algorithms such as Adam [42], deep learning methods effectively escape saddle points and local minima, achieving advanced solutions. Secondly, the end-to-end learning strategy eliminates the need for precise initialization when using deep learning methods to compute holograms. Thirdly, designing the loss function based on specific objectives can guide the network toward convergence in desired directions. Therefore, the deep learning method provides holography-based TPL with a new technical pathway and expanded possibilities. To apply deep learning, the hologram generation inverse problem is formulated as an optimization problem:

$$\underset{H}{\text{argmin}}L\left(T,\;{\hat{I}}^{k}\left(H\right)\right),$$ (1)

where *H* represents the hologram, *T* represents the fabrication target, $\hat{I}$ represents the modulated light field intensity in simulation, and *k* is the nonlinear coefficient of photoresist. Deep learning utilizes gradient descent algorithms to minimize the loss function, thereby updating network parameters and solving the nonconvex optimization problem. A customizable loss function L enhances the algorithm’s flexibility.

**Fig. 1.**
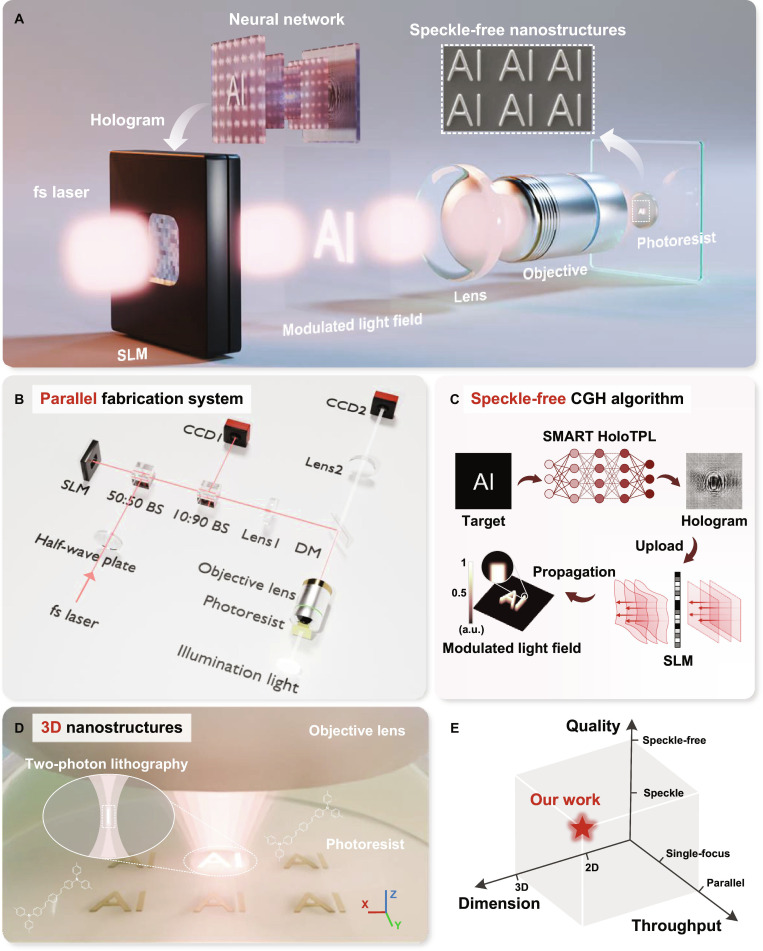
Schematic of SMART HoloTPL. (A) Fundamental principle of speckle-free nanofabrication by SMART HoloTPL. (B) Parallel fabrication system of SMART HoloTPL based on holographic light fields. (C) SMART HoloTPL utilizes deep learning to generate speckle-free holograms. (D) Fabrication of 3D nanostructures by 2-photon polymerization of custom-developed photoresist. (E) Performance comparison in terms of fabrication quality, throughput, and dimension, highlighting the advantages of SMART HoloTPL in achieving parallel, speckle-free 3D nanofabrication.

**Fig. 2.**
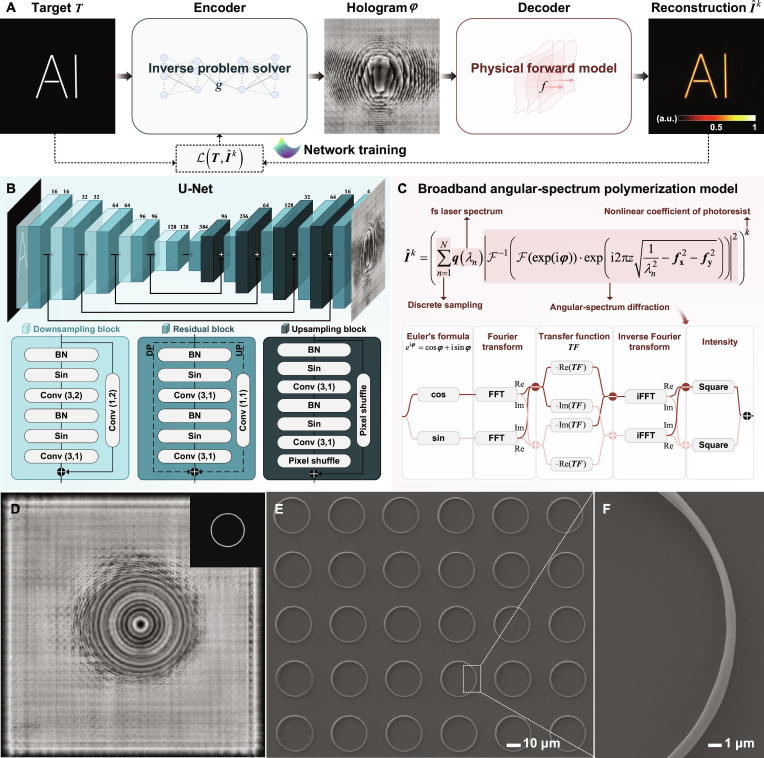
Complete pipeline of SMART HoloTPL: from physical modeling to hologram generation and fabrication. (A) Solving an inverse problem of hologram generation based on a physical forward model. (B) Neural network architecture. Conv(*a*,*b*), convolutional layer with kernel size a×a and stride *b*; DP, downsampling path; UP, upsampling path. (C) Broadband angular-spectrum polymerization model. The calculation of the model is based on Euler’s formula. (D) Target image and hologram of a ring structure. (E) SEM of ring structures. (F) Zoomed-in view.

Here, we propose a deep learning method for parallel, speckle-free 3D nanofabrication, as shown in Fig. 1. To overcome the lack of ideal holograms as ground truth, we employ a physical forward model to guide network training directly, referred to as model-driven deep learning. The network framework adopts an autoencoder, as shown in Fig. 2A. The encoder is a U-Net, acting as an inverse problem solver to predict holograms from target inputs. The decoder is the physical forward model for simulating the reconstructed light field distributions. The neural network is trained without ground truth by minimizing the difference between targets and reconstructions. This framework fully utilizes the network’s inverse problem-solving capabilities while enhancing the interpretability of network training.

### Broadband angular-spectrum polymerization model

From the perspective of energy efficiency and information capacity of modulators, phase-only liquid-crystal-on-silicon (LCoS) SLM is employed to modulate the light fields. Therefore, the inverse problem is further specified as the generation of a phase-only hologram (POH) φ. Compared to the Fourier transform, diffraction propagation offers a more effective approach for modulating 3D light fields. ASM is widely utilized for diffraction calculations due to its advantages, including no paraxial approximation and the invariance of the modulated target size with propagation distance [43]. Here, we develop a physical forward model based on ASM to simulate the physical process from the SLM to the modulated light field. ASM is a rigorous scalar diffraction calculation method based on the plane wave solution of the Helmholtz equation. Its core principle is to treat any arbitrary transverse light field as a linear superposition of plane wave components propagating in different directions, with each component evolving independently along the propagation axis. Let $U\left(x,\;y\right)$ denote the complex amplitude distribution on the *z* = 0 plane; its 2D Fourier transform is defined as:

$$U\left(f_{x},\;f_{y}\right)=\iint_{-\infty }^{\infty }U\left(x,\;y\right)\exp\left(-i2\pi \left(f_{x}x\;+\;f_{y}y\right)\right)\;\mathrm{dx}\;\mathrm{dy},$$ (2)

where $\left(f_{x},\;f_{y}\right)$ represents the transverse spatial frequencies. After each frequency component of the light field propagates a distance *z* along the axial direction in free space, its phase change is governed by the transfer function in the frequency domain:

$$\mathrm{TF}\left(f_{x},\;f_{y}\right)=\exp\left(i2\pi z\sqrt{\frac{1}{\lambda^{2}}-f_{x}^{2}-f_{y}^{2}}\right).$$ (3)

In this expression, the square root term corresponds to the spatial frequency component $f_{z}$ in the *z* direction. When $f_{x}^{2}+f_{y}^{2}\leq \frac{1}{\lambda^{2}}$, the light field at distance *z* can be obtained via the inverse Fourier transform:

$$\begin{array}{c} \begin{array}{c} \begin{array}{c} \begin{array}{cc} \hat{U}\left(x,\;y\right) & =\iint_{-\infty }^{\infty }U\left(f_{x},\;f_{y}\right)\mathrm{TF}\left(f_{x},\;f_{y}\right)\exp\left(i2\pi \left(f_{x}x+f_{y}y\right)\right)\;{\mathrm{df}}_{x}\;{\mathrm{df}}_{y}\\ & \;=F^{-1}\;\left(F\;\left(U\left(x,\;y\right)\right)\cdot \exp\left(i2\pi z\sqrt{\frac{1}{\lambda^{2}}-f_{x}^{2}-f_{y}^{2}}\right)\right), \end{array} \end{array} \end{array} \end{array}$$ (4)

where $F\left(\cdot \right)$ and $F^{-1}\left(\cdot \right)$ denote the Fourier transform and inverse Fourier transform operations, respectively. So the modulated light field can be obtained:

$$\hat{U}\left(x,\;y\right)=F^{-1}\;\left(F\;\left(\exp\left(i\varphi \right)\right)\cdot \exp\left(i2\pi z\sqrt{\frac{1}{\lambda^{2}}-f_{x}^{2}-f_{y}^{2}}\right)\right).$$ (5)

In practical numerical calculations, the fast Fourier transform (FFT) is commonly employed to enhance computational efficiency.

Due to the broadband nature of fs laser pulses, which results from the time-bandwidth product conservation [44], these pulses exhibit obvious multi-wavelength propagation effects. Therefore, we extend the classic ASM to ensure accurate modeling. Specifically, the fs laser spectrum is discretely sampled, and then the reconstructed intensities at the sampled wavelengths are summed [45]. The intensity of the modulated light field is given by:

$$\hat{I}=\sum_{n=1}^{N}q\left(\lambda_{n}\right){\left|F^{-1}\left(F\left(\exp\left(i\varphi \right)\right)\cdot \exp\left(i2\pi z\sqrt{\frac{1}{\lambda_{n}^{2}}-f_{x}^{2}-f_{y}^{2}}\right)\right)\right|}^{2},$$ (6)

where $q\left(\lambda_{n}\right)$ denotes the spectral density of the fs laser.

Additionally, to account for the nonlinear response of the photoresist, the summed intensity is raised to the power of its multi-photon coefficient *k*, yielding the model to constrain network training:

$${\hat{I}}^{k}={\left(\sum_{n=1}^{N}q\left(\lambda_{n}\right){\left|F^{-1}\left(F\left(\exp\left(i\varphi \right)\right)\cdot \exp\left(i2\pi z\sqrt{\frac{1}{\lambda_{n}^{2}}-f_{x}^{2}-f_{y}^{2}}\right)\right)\right|}^{2}\right)}^{k}.$$ (7)

To fully leverage existing real-valued network training resources, the proposed complex-valued model is decomposed into separate real and imaginary parts, facilitating effective gradient propagation via chain rule implementations. The specific calculation process is shown in Fig. 2C. The predicted complex amplitude distribution $e^{i\varphi }$ at the hologram plane is expressed based on Euler’s formula and split into a red real-part branch and a pink imaginary-part branch, which are then calculated individually. Since the Fourier transform, transfer function, and inverse Fourier transform produce complex-valued outputs, each branch undergoes further separation and recombination into real and imaginary parts. The final intensity is computed as the sum of squared magnitudes. This process is repeated across all sampled wavelengths to obtain the simulated reconstruction ${\hat{I}}^{k}$. The simulated reconstruction is subsequently used to calculate the loss function and update the learnable parameters of the network. This strategy ensures computational accuracy while maintaining compatibility with standard real-valued network training paradigms.

The robustness of the proposed polymerization model is analyzed as follows. First, owing to the layer-based approach, this model is compatible with any holographic design within each layer. This capability for arbitrary pattern modulation represents the core advantage of CGH. Furthermore, the angular-spectrum propagation model determines the modulation depth range and feature size. Specifically, the feature size is consistent with the pixel pitch of the SLM, and the modulation depth must be maintained within the range of

$$z\leq \mathrm{Mp}\sqrt{4p^{2}\lambda^{-2}-1},$$ (8)

where *M* and *p* are the pixel number and pixel pitch of the SLM, respectively [43].

### Model-driven deep learning for hologram generation

U-Net is a widely adopted convolutional neural network (CNN) featuring a symmetric downsampling–upsampling architecture with skip connections [46]. U-Net has demonstrated remarkable performance across various image-to-image tasks, such as instance segmentation [47,48], super-resolution [49], and image denoising [50]. To better address the POH generation problem, we designed the network architecture based on U-Net, as shown in Fig. 2B. To address the challenge of reconstruction accuracy, the periodic activation function sin enhances the high-frequency components of reconstructed light fields, thereby mitigating the loss of fine details during network training [51,52]. For the challenge posed by the large-scale hologram size (i.e., 1,080 × 1,080), 2 strategies are adopted. First, the network depth is appropriately increased, and residual blocks are incorporated to mitigate the vanishing gradient problem [53]. Second, instead of conventional pooling and unpooling, convolution with a stride of 2 and subpixel convolution are employed for downsampling and upsampling, respectively, introducing additional learnable parameters [54].

Network training is implemented in a self-supervised manner, overcoming limitations imposed by non-ideal ground truth in terms of quality, scale, and generalization. Without explicit supervision, the loss function directly influences convergence and must be carefully designed based on physical priors. Here, we construct a composite loss function:

$$L=\tau L_{\mathrm{MSE}}\left(T,\;{\hat{I}}^{k}\right)+\left(1-\tau \right)L_{\mathrm{TV}}\left({\hat{I}}^{k}\right),$$ (9)

where the mean squared error (MSE) term ensures the fidelity and intensity of the modulated light field; the total variation (TV) regularization term enhances the uniformity of the modulated light field; and τ is an adjustable parameter for the balance of 2 terms. After training, holograms with speckle-free reconstructions can be obtained.

### Speckle-free nanofabrication with high throughput

The optical setup of the SMART HoloTPL system is shown in Fig. 1A. A regenerative fs laser amplifier with gigawatt-level peak power delivers 4 mJ per pulse to support the parallel fabrication. The fs laser is modulated by an SLM loaded with holograms calculated by the deep learning method. To compensate for system-induced wavefront aberrations of the SLM, the phase response is calibrated using a wavefront sensor and corrected via Zernike polynomial fitting, leading to improved intensity uniformity of the reconstructed light fields (Fig. S3). After propagating over the preconfigured distance, the modulated light field is obtained. Next, the modulated beam enters a 4-f relay system, which demagnifies and projects the nanoscale light field into the photoresist for polymerization. CCD1 and CCD2 monitor the real-time reconstructed light field and the in situ fabrication process, respectively.

The photoresist is custom-developed for compatibility with the ultrahigh-peak-power light source, incorporating photo-initiators with large absorption cross-sections and optimized polymer matrix components to ensure lithographic performance [23]. The photoresist exhibits a broad dynamic exposure range of 12.46 and, once cured, a refractive index of 1.52. Its formulation consists of 0.2 wt % 1,4-bis[4-(di-p-tolylamino)styryl]benzene photo-initiators, together with 4,4′-(4,4′-isopropylidenediphenoxy)-bis-(phthalic anhydride) (BPADA; 68 wt %) and pentaerythritol triacrylate (PETA; 32 wt %) as the monomers to enhance the mechanical strength of the photoresist.

**Fig. 3.**
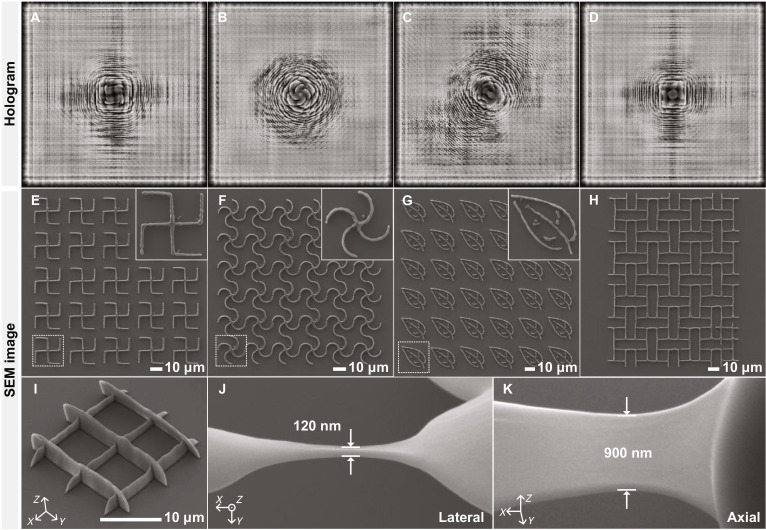
Various SMART HoloTPL-printed structures. Holograms (A to D) and corresponding SEM images (E to H) of chiral photonic crystals and leaf-like motifs. (I) 3D woodpile. (J) Lateral resolution. (K) Axial resolution.

Leveraging the algorithm’s high design flexibility and the photoresist’s superior photopolymerization sensitivity and mechanical robustness, SMART HoloTPL achieves high-resolution fabrication across diverse geometries. First, a simple ring structure with 560-nm lateral width was fabricated by a single exposure, as shown in Fig. 2E and F, yielding a smooth feature free of speckle artifacts. We then demonstrate SMART HoloTPL’s high-throughput capabilities with the holograms and corresponding scanning electron microscopy (SEM) images of various microstructures in Fig. 3. Planar arrays of chiral photonic crystals can be fabricated by SMART HoloTPL. Each structure exhibits uniform periodicity and high fidelity over regions exceeding 10,000 μm^2^, highlighting the robustness and reproducibility of SMART HoloTPL. The leaf-like structure in Fig. 3G further verifies the high flexibility of SMART HoloTPL for fabrication target selection. Nonperiodic structures, like Fig. 3H, can also be fabricated efficiently by conveniently switching holograms to vary the polymerization positions. Figure 3I shows a 2-layer woodpile architecture with an interlayer spacing of 3 μm, demonstrating the capability for 3D fabrication. Notably, the large-area arrays in Fig. 3 are formed by laterally stitching unit structures generated using the same or switched holograms, implicitly demonstrating good repetition consistency and robustness against typical environmental perturbations under standard laboratory conditions.

SMART HoloTPL achieves subdiffraction-limited fabrication with lateral and axial resolutions of 120 and 900 nm, respectively. The relatively low axial resolution primarily arises from the limited pixel size of the SLM. Smaller pixels can support a broader angular spectrum, enabling a tighter depth of focus and thus achieving higher axial resolution. In our system, up to 2,000 voxels can be fabricated with high quality in a single exposure. Accordingly, most demonstrated patterns were fabricated using approximately 1,800 to 2,000 voxels per exposure. Here, the voxel is defined based on the effective pixel pitch of the projected pattern under the 40× objective (corresponding to 200 nm), consistent with conventional TPL metrics. In the angular-spectrum-based model, the computational pixel size matches the SLM pixel pitch (8 μm), which directly maps to the defined voxel size after demagnification by the objective, ensuring consistency between modeling and experiment. Notably, volumetric layer projection further avoids minimum inter-focal distance constraints and reduces crosstalk, enabling uniform voxel formation in a single exposure. The exposure repetition rate is set to 60 Hz by the SLM, resulting in a rate of 120,000 voxels/s, which can be further improved with a higher refresh-rate SLM.

### Comparison with GS algorithms

**Fig. 4.**
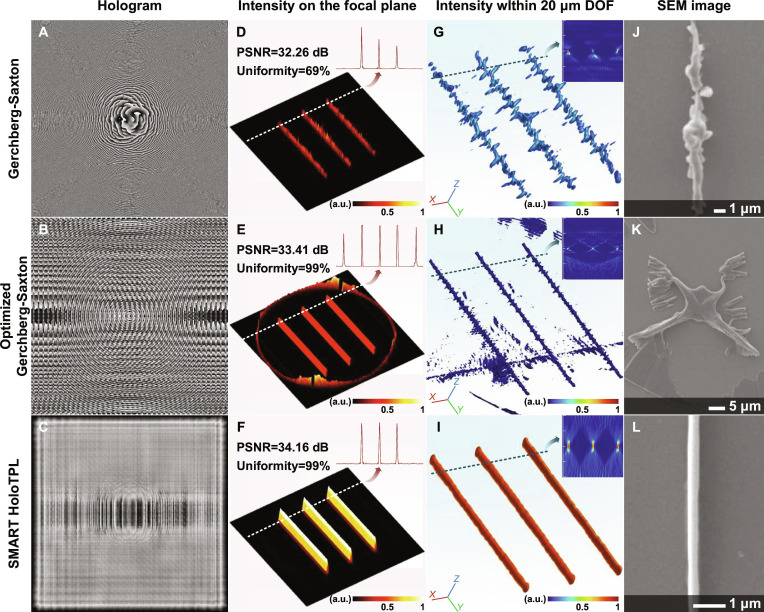
Conventional hologram generation algorithms versus SMART HoloTPL in terms of reconstruction accuracy and fabrication quality. (A to C) Generated holograms using GS, optimized GS, and SMART HoloTPL. (D to F) Corresponding simulated intensity distributions on the focal plane. (G to I) 3D simulation results of the modulated light fields within 20-μm depth of focus (DOF). (J to L) SEM images of the fabricated structures.

To benchmark SMART HoloTPL, we compare it against the GS algorithm and another frequently used modified GS algorithm [26]. Figure 4A to C shows the calculated holograms by the 3 algorithms. Their simulated focal-plane intensity distributions appear in Fig. 4D to F, immersive 3D light field simulations in Fig. 4G to I, and corresponding fabrication results in SEM images in Fig. 4J to L. The uniformity of the reconstructed light field is used to quantitatively evaluate the speckle reduction performance of different algorithms.

The hologram generated by the GS algorithm, as shown in Fig. 4A, is initialized with a random phase and converges after multiple iterations. However, the intensity on the focal plane is significantly affected by speckle noise, leading to only 69% uniformity, which gives rise to the surface roughness observed in the simulation shown in Fig. 4G. The experimental fabrication shown in Fig. 4J matches the simulation well, exhibiting broader and irregular linewidths compared to the designed target.

The modified GS algorithm based on a smooth initial phase achieves 99% uniform intensity distribution on the focal plane, enabling speckle-free 2D fabrication based on spin coating. However, the uniformity deteriorates significantly away from the focal plane, as shown in Fig. S6. Therefore, the intensity distribution within 20-μm depth of focus is still rough, as shown in Fig. 4H. Moreover, the light field exhibits a significant intensity concentration above the focal plane. From the corresponding fabrication result in Fig. 4K, we can observe that the polymerized structures located above the focal plane obstruct and distort the target’s polymerization, making it difficult to achieve immersive fabrication for 3D structures.

In comparison, SMART HoloTPL produces a uniform intensity distribution without speckle artifacts both on the focal plane and within a certain depth, as shown in Fig. 4F and I and Fig. S6. SMART HoloTPL achieves the highest peak signal-to-noise ratio (PSNR) and uniformity, demonstrating its fidelity advantages. As shown in Fig. 4L, the fabrication result of SMART HoloTPL demonstrates morphological features and surface smoothness consistent with the simulation result. In addition to SEM characterization, atomic force microscopy measurements were performed to quantitatively assess surface roughness and dimensional fidelity. The fabricated structures exhibit low surface roughness and good dimensional consistency (Fig. S8). Critically, the fabricated line structures preserve their designed width over the entire axial range, simultaneously confirming high fabrication throughput and true volumetric uniformity.

## Discussion

In summary, we analyzed the origins of speckle noise in alternating projection algorithms and proposed a model-driven deep learning approach to generate speckle-free holograms. SMART HoloTPL is capable of high-throughput 3D nanofabrication while maintaining submicrometer feature sizes, attaining a lateral resolution of 120 nm and a fabrication rate of 120,000 voxels/s and provides an effective solution for extending TPL to applications beyond laboratory prototyping.

While single-exposure fabrication of complex 3D structures remains challenging, this limitation mainly arises from maintaining a uniform axial light field distribution and from the finite depth of focus imposed by high-numerical aperture objectives. SMART HoloTPL therefore adopts line-based fabrication units with well-controlled *z*-axis intensity profiles, and complex structures are constructed through multiple exposures of these units. While this introduces a trade-off in fabrication efficiency for highly complex geometries, it preserves fabrication uniformity and robustness within the intrinsic constraints of TPL. Moreover, further integration of deep learning with physics and a deeper understanding of nonlinear polymerization mechanisms may enable superior outcomes. In addition, constraining network training with real physical processes rather than models offers a promising path to higher-resolution 3D nanofabrication. Beyond TPL, this inverse problem-solving framework readily applies to broader fabrication processes.

## Materials and Methods

### Optical setup

The fs regenerative laser amplifier has an 800-nm central wavelength, 100-fs pulse width, and 10-nm spectral width. Because the SLM is polarization-sensitive, a half-wave plate was used to change the illuminating light into transverse linearly polarized light to reduce the effect of zero-order light that cannot be modulated. The SLM has an 8-bit gray level, 8-μm pixel pitch, and 1,920 × 1,080 pixels. The diffraction efficiency of SLM can be adjusted by look-up table (LUT). It is worth noting that the surface normal of the beam splitter (BS) after SLM needs to be at a small angle to the optical axis to avoid the effect of interference fringes on the reconstructions caused by the reflected light from the surface before and after the BS. To meet the Nyquist sampling theorem, here the reconstruction distance is set as 160 mm. The objective lens is a 40× oil immersion lens.

### U-Net architecture

Five downsampling blocks and 5 corresponding upsampling blocks are used. Subpixel convolution includes a convolutional layer to increase the channel number and a pixel shuffle layer to rearrange the tensor from H×W×2C to 2H×2W×C/2. The grayscale image dataset DIV2K is used for network pretraining.

### Loss function

MSE and TV are utilized as loss functions to constrain the network training. MSE is formulated as

$$\mathrm{MSE}\left(A,\;\hat{A}\right)=\frac{1}{\mathrm{MN}}\sum_{i=1}^{M}\sum_{j=1}^{N}{\left(A_{i,j}-{\hat{A}}_{i,j}\right)}^{2}.$$ (10)

TV is formulated as

$$\mathrm{TV}\left(A\right)=\sum_{i=1}^{M}\sum_{j=1}^{N}\left(\left|A_{i+1,j}-A_{i,j}\right|+\left|A_{i,j+1}-A_{i,j}\right|\right).$$ (11)

### Quantitative metric

PSNR and uniformity are utilized as evaluation indicators to assess the accuracy of simulated reconstructions quantitatively. PSNR is formulated as

$$\text{PSNR}\left(A,\;\hat{\text{A}}\right)=10\;\log_{10}\left(\frac{{\text{MAX}}^{2}}{\mathrm{MSE}\left(A,\;\hat{A}\right)}\right),$$ (12)

where MAX is the maximum pixel value. Uniformity is formulated as

$$\begin{array}{c} \begin{array}{c} \text{Uniformity}=1-\frac{\sigma }{\mu },\\ \;\mu =\frac{1}{\mathrm{MN}}\sum_{i=1}^{M}\sum_{j=1}^{N}A_{i,j},\;\sigma =\sqrt{\frac{1}{\mathrm{MN}}\sum_{i=1}^{M}\sum_{j=1}^{N}{\left(A_{i,j}-\mu \right)}^{2}}. \end{array} \end{array}$$ (13)

## Data Availability

The algorithms and data supporting this work are available at https://github.com/THUHoloLab/SMART_HoloTPL.
