# Supplementary material for: Model-Driven Deep Learning Enables Speckle-Free Holography for 3D Parallel Nanofabrication
Source: Research (Wash D C). 2026 Apr 14;9:1159. doi: 10.34133/research.1159 (PMC13077129; doi:10.34133/research.1159)
Supplement: Supplementary 1 — Supplementary Text Figs. S1 to S8 [file research.1159.f1.docx]

Supplementary Materials for

**Model-driven deep learning enables speckle-free holography**

**for 3D parallel nanofabrication**

Kexuan Liu *et al.*

*Corresponding author. Email: [clc@tsinghua.edu.cn](mailto:clc@tsinghua.edu.cn), [scchen@mae.cuhk.edu.hk](mailto:scchen@mae.cuhk.edu.hk)

**This PDF file includes:**

Supplementary Text

Figs. S1 to S8

Supplementary Text

**Nanofabrication system configuration**

A detailed optical layout of the high-throughput TPL nanofabrication system is shown in Supplementary Fig. S1. The light source is a Ti:sapphire regenerative amplifier (Spitfire Pro, Spectra-Physics) operating at a central wavelength of 800 nm, a repetition rate of 1 kHz, a pulse duration of 100 fs, and an average power of 4 W. Laser power is tuned using a half-wave plate in combination with a polarizing beam splitter (PBS). A Galilean beam expander, consisting of a plano-concave (f = -100 mm) and a plano-convex lens (f = 250 mm), expands the beam to match the aperture of the spatial light modulator (SLM). The SLM (PLUTO-2.1-NIR-113, HOLOEYE) features phase-only modulation, a resolution of 1920×1080 pixels, 8 µm pixel pitch, and operates at 60 Hz. The modulated target light field forms 160 mm downstream of the SLM, and a spatial filter blocks undesired diffraction orders. A 10:90 beam splitter (BS) directs 10% of the optical power to Camera 1 (MV-CU020-90GM, Hikvision) for real-time monitoring. The remaining 90% of the beam is relayed by a 4f system, formed by a plano-convex lens (f = 200 mm) and a high numerical aperture (NA) oil-immersion objective (Nikon CFI S Fluor 40× Oil, NA = 1.3, WD = 0.24 mm), to project the demagnified target light field into the photoresist for nanofabrication. The objective is immersed directly in a drop of photoresist to improve fabrication resolution.

The glass substrate with photoresist is mounted on a precision six-axis stage (H-811.I2, Physik Instrumente) to minimize stitching errors and to compensate for tip-tilt errors during large-area patterning. To enable in situ monitoring of the fabrication process, a trans-illumination microscope is integrated into the optical path and shares the objective. A dichroic mirror (DM) reflects the 800 nm modulated light field into the objective while transmitting visible LED illumination, allowing simultaneous microscopic imaging via Camera 2 (MV-CS060-10UM-PRO, Hikvision). The captured images of the modulated light field by Camera 1 and the fabrication process by Camera 2 are shown in Supplementary Fig. S2a and S2b, respectively.

The repeatability and stability of the nanofabrication process are primarily determined by the overall system configuration and operating conditions. In the present setup, the femtosecond laser amplifier provides stable output under standard laboratory conditions, ensuring consistent exposure during repeated fabrication. The positioning accuracy of the six-axis stages is ~100 nm, which is sufficient for the feature sizes and lateral stitching demonstrated in this work. In addition, the optical setup is mechanically stable, and the holographic projection implemented on the SLM exhibits stable phase and intensity response over time. Together, these factors enable reliable and repeatable fabrication under the reported experimental conditions.

**Calibration of the spatial light modulator**

The surface flatness of the liquid crystal panel in the SLM plays a critical role in determining the uniformity of both the modulated light fields and the fabrication quality, particularly under the stringent requirements of sub-diffraction-limit fabrication. To quantify optical aberrations introduced by the SLM, we employed a wavefront sensor (WFS40-7AR(/M), Thorlabs) to measure the phase of light directly reflected from the SLM without modulation. Based on the measured aberrations, phase compensation was performed using Zernike polynomial fitting on SLM dominated by vertical/horizontal astigmatism terms ($Z_{2}^{2}$). This calibration enables experimental results that closely match the simulations, shown in Fig. S3b.

**Design of the photoresist**

To ensure compatibility with high-peak-power fs laser, a custom photoresist was developed based on our previous work (*14*). The formulation involved dissolving 0.2 wt% of a photo-initiator (CAS No. 55035-43-3) into a monomer mixture consisting of 32 wt% pentaerythritol triacrylate (PETA) and 68 wt% 4,4’-(4,4’-isopropylidenediphenoxy)-bis-(phthalic anhydride) (BPADA). The molecular structure of the photo-initiator is shown in Fig. S4. The components were thoroughly mixed using ultrasonic agitation at 55 °C for one hour to ensure homogeneity.

As reported in our earlier study (*14*), the nonlinear coefficient$N$of the photoresist was derived from the empirical relationship:

$$D\propto\tau RE_{p}^{N},$$

where$D$is the effective laser dose,$\tau$is the exposure time,$R$is the laser repetition rate, and$E_{p}$is the pulse energy. The calculated nonlinear coefficient was$N=6.65$, indicating a highly nonlinear two-photon polymerization behavior.

Furthermore, the photoresist demonstrated a broad dynamic range of 12.46, calculated from the polymerization and damage thresholds. Specifically, the polymerization threshold was measured at 1.27 nJ, while the damage threshold was 17.10 nJ. This wide operating window ensures precise voxel definition and minimizes structural damage during high-resolution laser writing.

**Hologram computation**

All holograms in this work were computed on a workstation equipped with an Intel Xeon Gold 6148 CPU, 512 GB RAM, and an NVIDIA A100 Tensor Core GPU. Calculations were conducted in MATLAB R2024b with GPU acceleration.

The Gerchberg-Saxton (GS) algorithm is a classical iterative method for hologram generation. It alternates between the hologram and target planes with amplitude constraints at each iteration. Starting from a randomly initialized phase , the complex amplitude field at the hologram plane is given by:

where$A_{h}$is the amplitude at the hologram plane and set as a uniform distribution. The field is then propagated to the target plane:

where the propagation modelcould be Fourier transform, Fresnel diffraction, or angular-spectrum diffraction. At the target plane, the amplitude is replaced by the desired amplitude$A_{t}$, and then the field is propagated back to update the phase at hologram plane:

This process is iterated until convergence is achieved at the target plane. Despite its simplicity and widespread use, the GS algorithm often suffers from speckle noise and slow convergence.

In 2016, an efficient optimized GS algorithm known as the Mixed Region Amplitude Freedom (MRAF), was introduced to enhance the quality of reconstructed light fields (*17*). The MRAF algorithm partitions the target plane into a signal region and a background region. Constraints are applied to the signal region:

$$A'_{t}(\mathbf{r})=\left\{\begin{matrix} \alpha A_{t}(\mathbf{r})+(1-\alpha)\alpha|U_{t}(\mathbf{r})|, & \mathbf{r}\in\text{signal region} \\ |U_{t}(\mathbf{r})|, & \mathbf{r}\in\text{background region} \end{matrix}\right.$$

where$0<\alpha<1$is an adjustable weighting factor. Furthermore, a smooth initial phase is selected to mitigate speckle noise.

Compared to the above iterative algorithms, model-driven deep learning enables faster hologram prediction. In contrast to data-driven deep learning, model-driven deep learning overcomes the performance limitations imposed by ground-truth dataset. By directly solving the inverse problem rather than learning the mapping between inputs and target datasets, the neural network successfully predicts speckle-free holograms. Furthermore, the network used in the proposed SMART HoloTPL is specifically designed for micro/nanofabrication tasks to enhance fabrication uniformity.

Supplementary Fig. S5 presents a comparison of the computational efficiency and reconstruction quality of GS, MRAF, and SMART. SMART demonstrates improved performance over conventional algorithms in both aspects and can further enhance reconstruction quality through transfer learning. In comparison, GS tends to stagnate at local minima, whereas MRAF involves longer computational time.

Supplementary Fig. S6 presents a comparison of the reconstructed light field uniformity obtained by the GS, MRAF, and SMART algorithms at different defocused positions. SMART maintains exceptionally high and stable uniformity across the entire axial range. In contrast, GS and MRAF exhibit pronounced variations, with significant degradation in uniformity away from the focal plane, leading to speckle-induced noise and rough surface features on the fabricated structures.

**Fabrication and characterization**

A soda lime glass substrate was thoroughly cleaned to remove surface contaminants and oxides. The cleaning procedure consisted of sequential ultrasonic baths: 20 minutes in a glass cleaning fluid, 10 minutes in deionized (DI) water, and 8 minutes in isopropyl alcohol (IPA), followed by drying in an oven at 80°C for two hours.

After cleaning, a drop of photoresist was then dispensed on the substrate, and the designed structures were directly fabricated by projecting holographic light fields into the drop, without the need for spin coating and enabling true 3D fabrication, as shown in Fig. S7.

Following exposure, the sample was developed in propylene glycol monomethyl ether acetate (PGMEA) for 10 minutes, rinsed in IPA for an additional 10 minutes, and dried under ambient conditions.

Optical images were captured with a COSSIM CMY-310 microscope. SEM was conducted using a JEOL JSM-7800F field-emission microscope at an acceleration voltage of 5 kilovolts with a tilted sample stage. Prior to SEM imaging, a thin platinum layer was sputter-coated onto the sample using an Edwards sputtering machine to enhance surface conductivity.

**3D visualization of simulated fabrication results**

The simulated fabrication results shown in Fig. 4 were obtained by intensity calculations followed by thresholding. Specifically, the intensity distribution within a 20 µm depth of focus was calculated layer by layer using the angular spectrum method, generating a 4D XYZ-Intensity data. This data was then visualized using MATLAB’s Volume Viewer app. Finally, by defining an appropriate transparency map to simulate the polymerization threshold in two-photon polymerization, the simulated fabrication result was obtained. The simulation accuracy has been experimentally confirmed, supporting its general applicability for predictive modeling prior to a wide range of two-photon lithography tasks.

**AFM characterization of fabricated nanostructures**

To quantitatively evaluate the fabrication quality beyond SEM imaging, atomic force microscopy (AFM) was performed on a representative uniformly printed nanoline fabricated on a glass substrate. AFM measurements (Supplementary Fig.S8) reveal a low surface roughness of the printed line, with an RMS roughness of 9.452 nm and an arithmetic roughness Ra of 4.643 nm, indicating a smooth surface profile at the nanometer scale. Cross-sectional height profiles extracted at multiple positions along the line show consistent line width and height, demonstrating good dimensional fidelity and uniformity. These quantitative results confirm that the proposed holographic fabrication approach enables precise and uniform nanoscale feature generation.


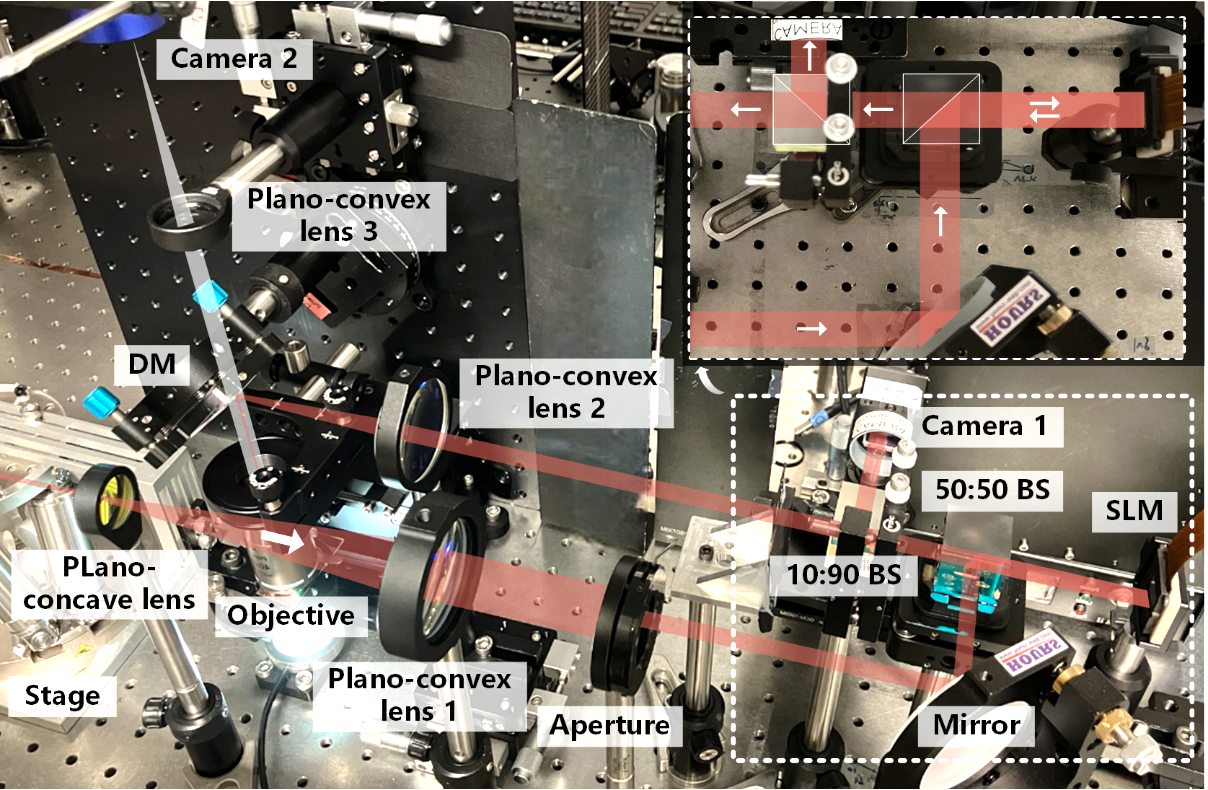


Fig. S1. Photograph of the nanofabrication system for SMART HoloTPL.


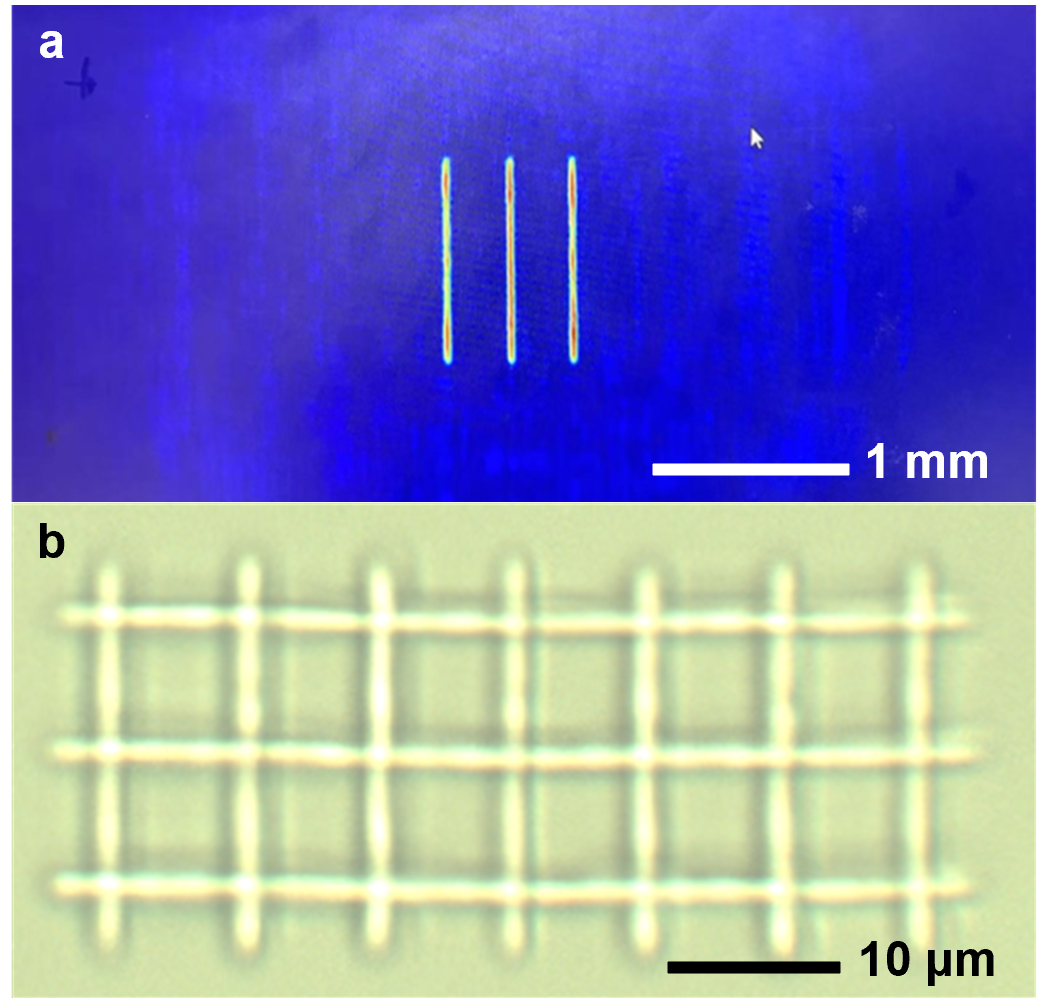


Fig. S2. (a) In-situ monitoring of the modulated light field. (b) In-situ monitoring of the fabrication process.


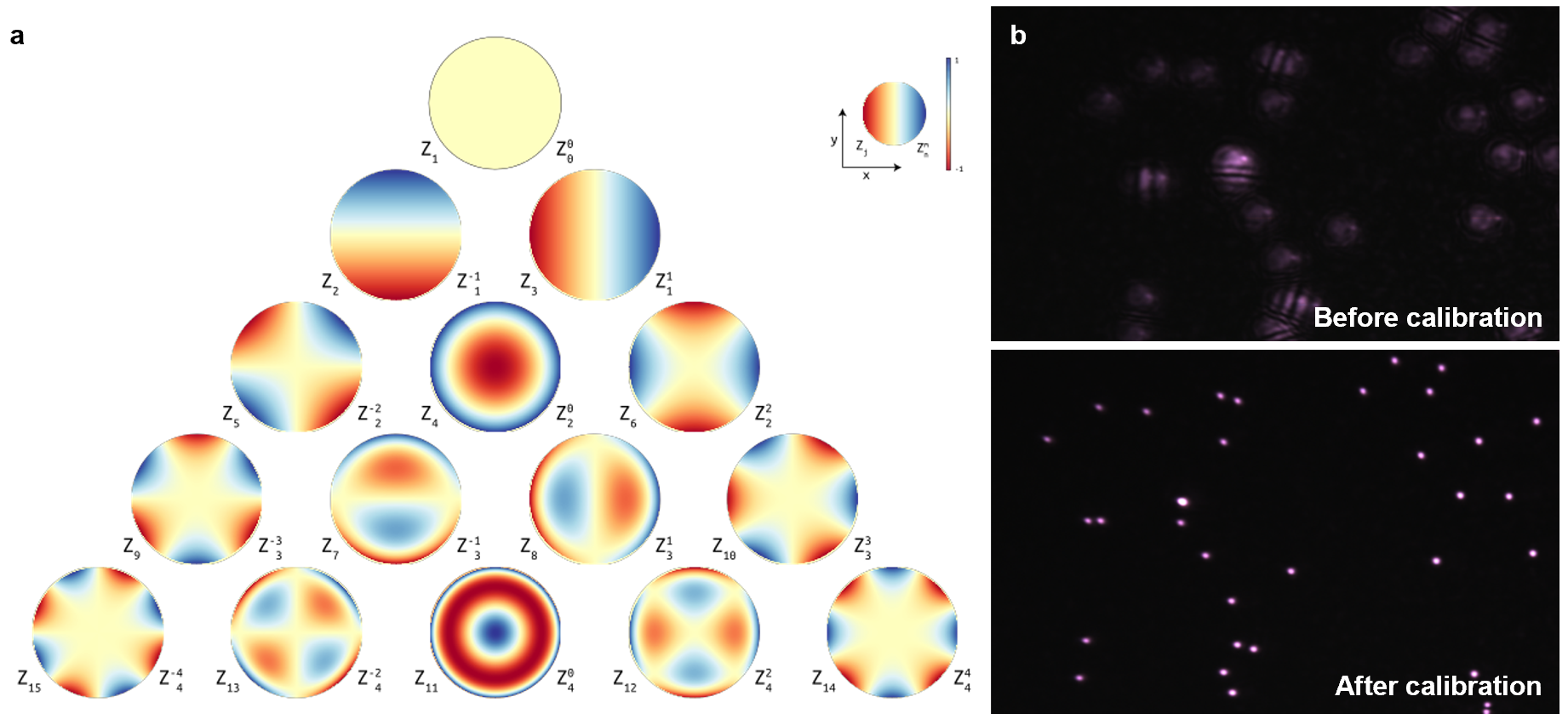


Fig. S3. (a) Zernike polynomials. (b) Multi-focus light field before and after calibration.


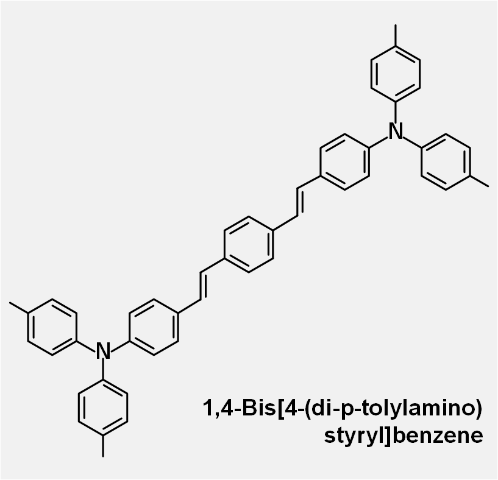


Fig. S4. Molecular structure of the initiator.

**
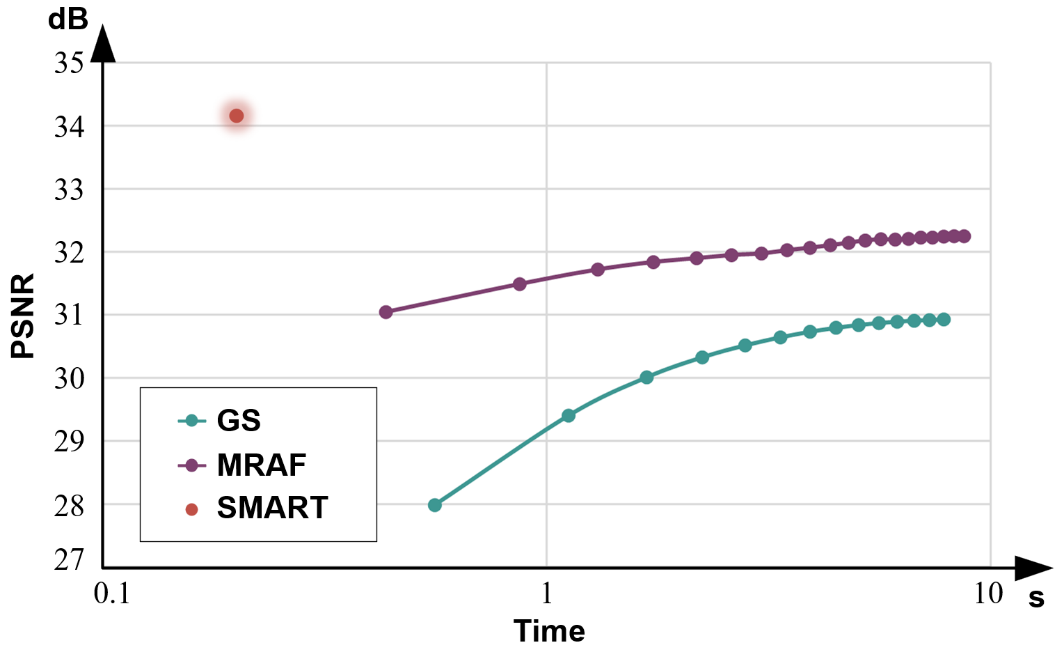
**

Fig. S5. Comparison of computational efficiency and reconstruction quality of GS, MRAF, and SMART.

**
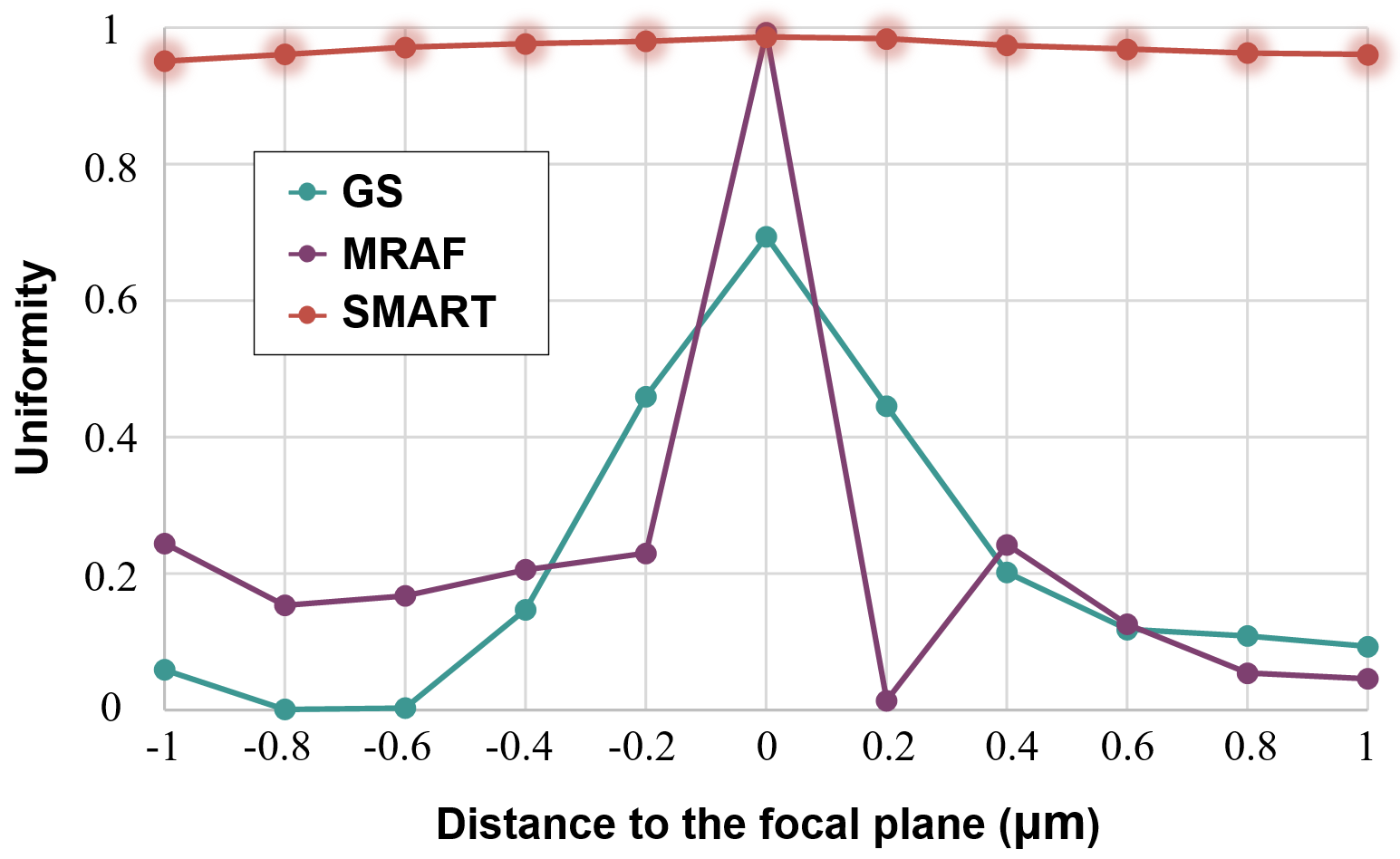
**

Fig. S6. Comparison of the uniformity of reconstructed light fields by GS, MRAF, and SMART at different defocused positions.


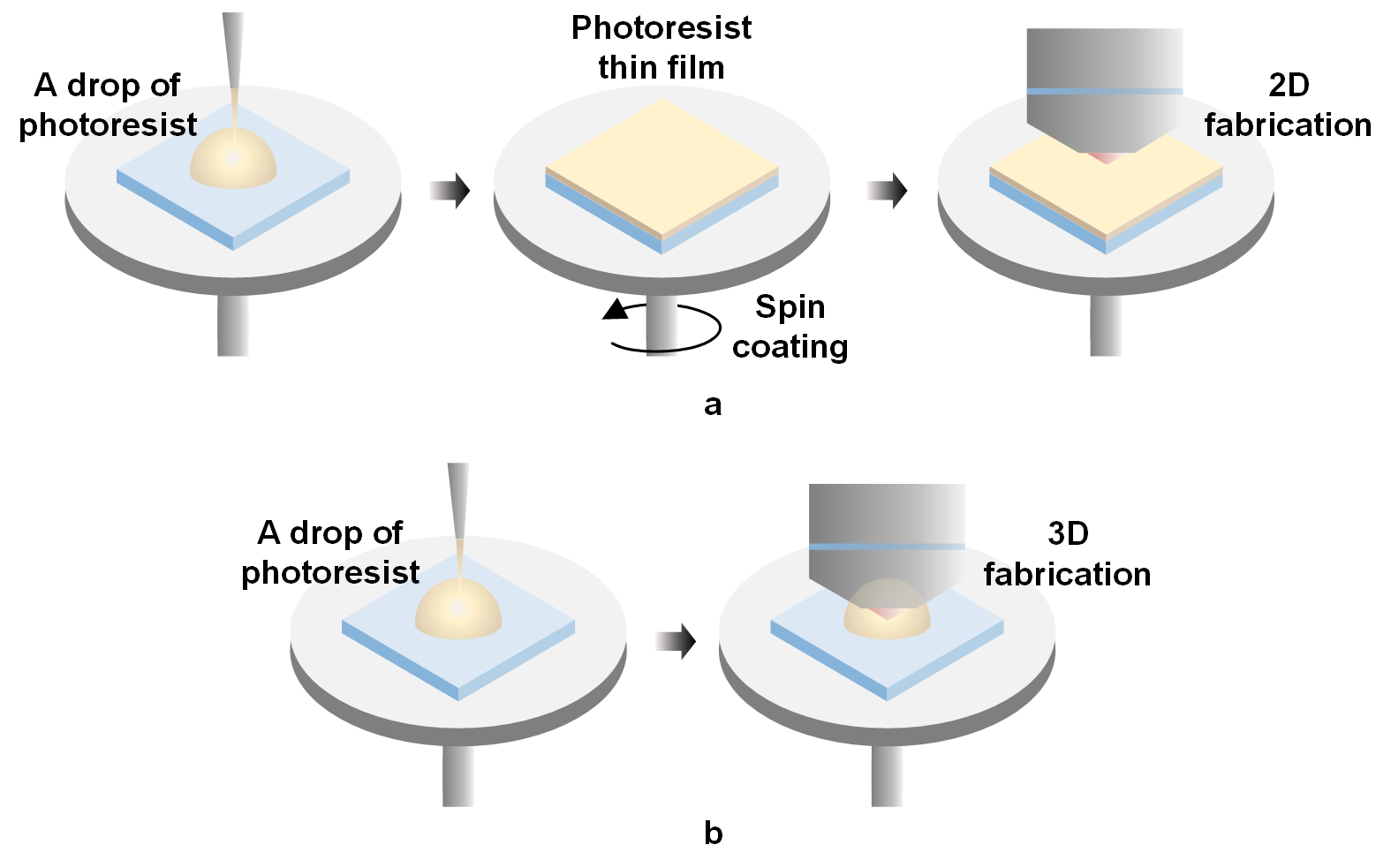


**Fig. S7. (a) Schematic of 2D fabrication based on a spin-coated photoresist thin film. (b) Schematic of 3D fabrication inside a drop of photoresist.**

**
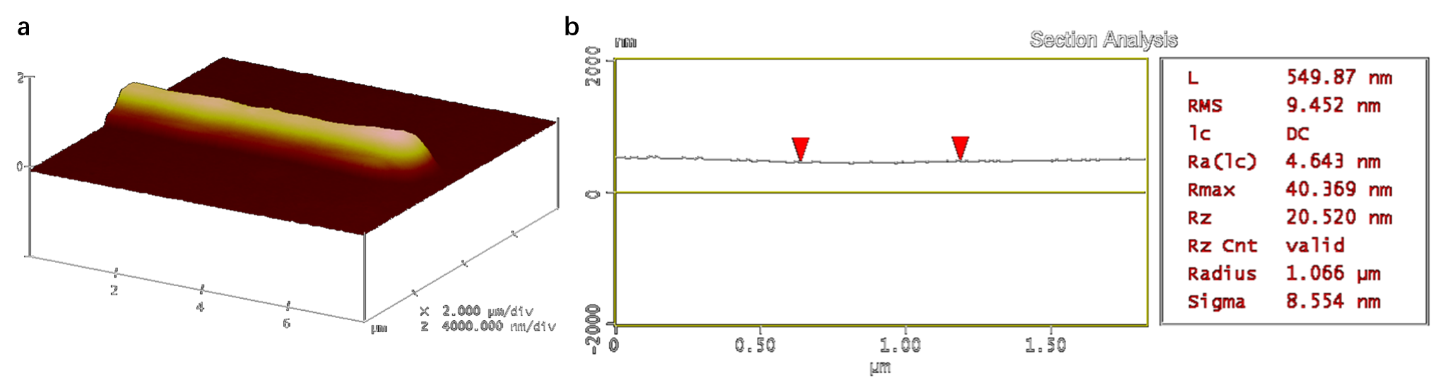
**

Fig. S8. (a) AFM height map of the line structures. (b) Representative cross-sectional height profiles.
